# Traumatic Brain Injury and Olfaction: A Systematic Review

**DOI:** 10.3389/fneur.2014.00005

**Published:** 2014-01-22

**Authors:** Peter William Schofield, Tammie Maree Moore, Andrew Gardner

**Affiliations:** ^1^Neuropsychiatry Service, Hunter New England Mental Health, Newcastle, NSW, Australia; ^2^Centre for Translational Neuroscience and Mental Health, University of Newcastle, Newcastle, NSW, Australia

**Keywords:** brain injury, olfaction, anosmia, trauma, review

## Abstract

Traumatic brain injury (TBI) is a common condition that is often complicated by neuropsychiatric sequelae that can have major impacts on function and quality of life. An alteration in the sense of smell is recognized as a relatively common complication of TBI; however in clinical practice, this complication may not be sought or adequately characterized. We conducted a systematic review of studies concerned with olfactory functioning following TBI. Our predetermined criteria led to the identification of 25 studies published in English, which we examined in detail. We have tabulated the data from these studies in eight separate tables, beginning with Table 1, which highlights each study’s key findings, and we provide a summary/synthesis of the findings in the accompanying results and discussion sections. Despite widely differing methodologies, the studies attest to a high frequency of post-TBI olfactory dysfunction and indicate that its presence can serve as a potential marker of additional structural or functional morbidities.

## Introduction

Traumatic brain injury (TBI) is a common, potentially preventable cause of mortality, and major morbidity. Neuropsychiatric sequelae, including cognitive, behavioral, and psychiatric symptoms and signs, may be present to varying degrees following a TBI according to the premorbid characteristics of the patient, its nature and severity, and the time elapsed since the injury. When specifically sought, olfactory functioning disturbances are common following TBI and, if present, can have a significant impact on quality of life (1). Reduced appreciation of food, drink, and other smell-laden sensual experiences; loss of employment, when this depends on an intact sense of smell; and increased danger from environmental hazards (e.g., volatile agents/gas, fires, spoiled food) are among the most obvious potential consequences of post-TBI olfactory deficits.

The availability in recent years of standardized instruments for assessing olfaction has enabled researchers to investigate with greater precision and rigor the associations between TBI (and related phenomena) and altered olfactory functioning (2, 3). However, despite the functional relevance of olfactory impairments following TBI, systematic objective quantitative testing of olfaction is not, at least in our own clinical experience, routinely undertaken. In light of this and because the substantial and growing literature suggests that olfactory impairment is relatively common and clinically important, we thought that a systematic review would be timely.

In the course of this review, issues of high clinical relevance are raised that, as far as we are aware, have not recently been systematically examined within the same work including: what relationship exists between TBI severity and the risk for post-TBI olfactory impairment? How commonly does olfactory impairment arise following a TBI? What are the structural and functional correlates of post-TBI olfactory impairment? What is the prognosis for post-TBI olfactory impairment? What is the impact of post-TBI olfactory impairment on quality of life?

## Materials and Methods

The review was conducted in two stages. In stage 1, articles were retrieved via online database searching. The online databases of PsycINFO, MEDLINE, EMBASE, Scopus, and COCHRANE were searched. Keywords and combinations of these words were used to search the databases comprehensively: diffuse axonal injury, brain hemorrhage, intracranial hemorrhage, brain edema, penetrating head injuries, olfaction disorders, olfactory perception, olfactory bulb, olfactory pathways, olfactory mucosa, olfactory receptor neurons, TBI, olfaction, olfactory, head injury, sense of smell, brain injury, smell, odor/odor, hyposmia, anosmia, brain damage, brain concussion, cerebral concussion, concussion, and craniocerebral trauma. Articles were limited to those that were published in English-language journals from 1980 to August 2013 and to studies in humans.

**Figure 1:**
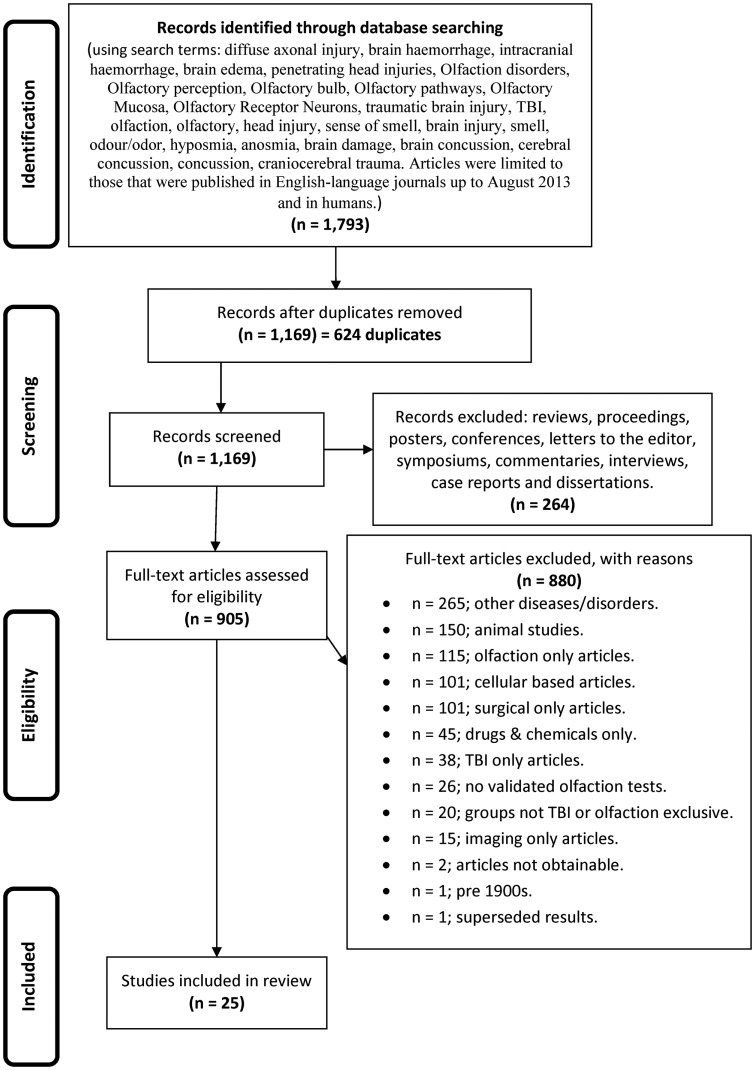
**The flow diagram depicting the search strategy, process, and exclusionary criteria by which the studies were selected for inclusion in this systematic review**.

During stage 2, the titles and abstracts of articles were reviewed to assess eligibility for inclusion in this review. Articles were regarded as relevant and warranting inclusion in the review if they were human studies, using validated olfactory testing methods in distinct TBI populations. Where there was uncertainty about whether a study should be included based on the review of the title and abstract, the full article was retrieved (see Figure 1 for article exclusion results).

## Data Extraction

Initially, one reviewer extracted data from the identified studies, including (1) participant demographics (TBI and control subjects), (2) characteristics of participants (TBI severity, duration of loss of consciousness (LOC), mechanism of injury), (3) olfactory testing paradigms (technique and data extraction), (4) time, elapsed since injury, (5) results of the study, and (6) study findings.

## Results

Our initial searches, based on the search strategies described in Figure 1, generated 1,793 hits. After duplicates were removed, we were left with 1,169 records, which were screened leading to removal of a further 264 records, leaving 905 full text articles that were further assessed for eligibility. As indicated in Figure 1, a further 880 were culled for the reasons listed.

### Scope, definitions, and methodology of the studies

Table 1 provides a brief overview of the main goals and findings of each of the 25 studies that we examined in detail (4–28) and the subsequent Tables 2–8 provide further detail regarding aspects of the methodology, sample characteristics, etc. The studies ranged broadly in terms of their principal focus (Table 1). Most of the studies included individuals who had sustained a TBI, regardless of the presence or otherwise of olfactory problems (5, 6, 9–12, 14–17, 19, 21–24, 26, 27) while in eight studies participants with TBI were selected for the presence of olfactory complaints or established olfactory impairment (4, 7, 8, 13, 18, 20, 25, 28). While most of the studies were cross sectional in design, several had an important longitudinal aspect (7, 9, 18, 26). The individual study sample sizes ranged from 5 individuals in the single qualitative study (25) to 367 (15). Only one of the studies was concerned with children with TBI specifically (14) (Table 2).

**Table 1:** **Main findings**.

Reference	Study design	Main purpose	Main findings
Parma et al. (4)	Cross sectional	To investigate implicit olfactory abilities in a group of anosmic	Evidence for implicit olfactory processing even when explicit olfactory testing suggests anosmia
		Individuals with TBI, matched with TBI patients with mild or no olfactory problems	
Neumann et al. (5)	Cross sectional	To investigate if olfaction is associated with affect recognition and empathy deficits after TBI	Olfactory deficits may be indicative of affect recognition impairments and reduced empathy
Charland-Verville et al. (6)	Cross sectional	To investigate if concussion(s) are associated with reductions in olfactory performance in athletes	No difference on olfactory measures (Sniffin Sticks) between controls with no history of concussion and those with one or multiple concussions. Longer delay since concussion was associated with worse performance on identification score. The investigators speculate that concussions may have a degenerative effect on olfactory function
Welge-Lussen et al. (7)	Longitudinal	To determine long-term recovery rates of post-traumatic olfactory disorders and evaluate whether lateralized disorder influences recovery	27% of patients improved at least six points on the composite score of the Sniffin Sticks over a more than 6 years interval. Lateralized olfactory dysfunction did not correlate with improvement rate
Gerami et al. (8)	Cross sectional	To examine the results of SPECT in anosmic subjects after closed head trauma, relative to no TBI normal controls, and look at the effect of olfactory stimulation on the SPECT results with orbitofrontal lobe the region of interest	Statistically different brain perfusion between cases and controls on all measures
Sigurdardottir et al. (9)	Longitudinal	To estimate the incidence of olfactory dysfunction across TBI severity (defined by GCS) and decision making deficits with regard to intracranial lesion-localization and laterality	Incidence of olfactory dysfunction was 22% at 3 months and 13.5% at 1 year. No association of olfactory dysfunction (as continuous variable) with TBI severity (although anosmia was). Verbal fluency (but not Iowa Gambling Task) was associated with olfactory tests
Vent et al. (10)	Cross sectional	Determine if boxers, as a group undergoing recurrent head trauma, demonstrate differences in olfactory performance relative to healthy controls	Boxers as a group performed significantly worse on the olfactory threshold and odor identification components of the Sniffin Sticks. There was an association of better olfactory performance with cushioning of the gloves
Fortin et al. (11)	Cross sectional	Compare the use of the UPSIT with the AST in TBI patients. Examine these data in relation to injury severity (GCS defined), depressive symptoms, awareness of olfactory impairment	The two tests were significantly correlated. Frontal lesions were associated with worse performance on olfactory tests. Mood and injury severity were not associated with olfaction. About 40% of individuals were unaware of olfactory deficits
Haxel et al. (12)	Cross sectional	Determine the incidence of olfactory dysfunction after head trauma using clinical, psychophysical, radiological, electrophysiological techniques. Efforts made to obtain unbiased estimates using sampling from a TBI cohort using a combination of inquiry, screening with the B-SIT, and follow up testing with the Sniffin Sticks	Estimated incidence of olfactory dysfunction after TBI was 12.8%. Olfactory dysfunction was related to skull based fractures and intracranial hemorrhage or hematoma
Rombaux et al. (13)	Cross sectional	To evaluate olfactory function with orthonasal and retronasal testing in patients with post-TBI olfactory loss and the relationship between residual olfactory function and olfactory bulb volume	There was an association between olfactory function and olfactory bulb volume and this was stronger for retronasal olfactory testing. Olfactory bulb volumes were lower in those with paraosmia than those without
Sandford et al. (14)	Cross sectional	To evaluate olfactory function in children with blunt head trauma	Children with blunt head injury may suffer post-traumatic olfactory impairment. There was an association between olfactory tests scores on the San Diego Children’s Odor Identification Test and TBI severity (when stratified “mild” or “moderate and severe” by GCS)
Green et al. (15)	Cross sectional	Investigate the relationship between brain injury severity and brain imaging abnormalities and olfactory test scores (AST), and neuropsychological test performance. All participants were involved in some form of compensation or medical disability claim. Individuals who failed symptom validity tests were excluded from analyses	Olfactory test scores predicted CT scan abnormalities, duration of PTA, GCS and LOC better than any of the neuropsychological scores singly or in combination
De Kruijk et al. (16)	Cross sectional	Determine the incidence of olfactory dysfunction 2 weeks after mTBI using an olfactory threshold test	Twenty-two percent of 111 patients had hyposmia and 4% had anosmia
Callahan and Hinkebein (17)	Cross sectional	To examine the performance characteristics of two forms of the University of Pennsylvania Smell Identification Test UPSIT (a 3-item version and the 40-item version) in a sample of individuals with TBI	Fifty-six percent of the sample had impaired olfaction on the full UPSIT, 40% of them were unaware of their deficits. Missing one item of the 3-item test related to a 2:1 likelihood of being anosmic. Nearly 20% of those who scored perfectly on the 3-item test scored in the anosmic range on the 40-item UPSIT
Fujii et al. (18)	Longitudinal	Investigate the changes in olfactory performance following a local injection of steroids into the nasal mucosa of patients with post-TBI olfactory impairment. Mean interval since injury was 6.6 months. No controls or placebo were included	On T&T Olfactometry, 35 and 23% improved on recognition and detection thresholds, respectively
Green and Iverson (19)	Cross sectional	To examine the relationship between exaggeration and scores on a test of olfactory discrimination in patients being assessed in connection with a claim for financial benefits	In patients with a TBI who failed tests of effort, there was no association between injury severity and total scores on the smell test. By contrast, in those who did pass a test of effort, there was an association between injury severity and olfactory performance
Yousem et al. (20)	Cross sectional	Define the primary sites of injury in patients with post-traumatic anosmia and hyposmia with MRI imaging and determine if these sites correlated with olfactory tests	The olfactory bulbs (89%), subfrontal lobes (61%), and temporal lobes (31%) showed the highest incidence of post-traumatic encephalomalacia. Left olfactory bulb and left tract volumes correlated with left and total UPSIT scores
Geisler et al. (21)	Cross sectional	Examine the relationship between olfactory event-related potentials (OERPs) and olfactory test and neuropsychological performance in a sample of individuals with TBI related olfactory change and controls	OERPs were related to olfactory test performance
Callahan and Hinkebein (22)	Cross sectional	To test the hypothesis that post-TBI anosmic patients do more poorly on measures of executive functioning and functional outcome than post-TBI patients without olfactory impairments	As a group, TBI patients with anosmia performed more poorly on a variety of executive function tasks and had greater disability than TBI patients without olfactory impairment
Doty et al. (23)	Cross sectional sub-group longitudinal	To examine olfactory function (and change in olfaction over time) and the influence of age, sex, TBI severity, time since TBI on this in patients with TBI who had presented initially with olfactory complaints. MRI brain imaging results were also examined in relation to olfactory symptoms and signs	Although there may be some improvement in symptoms and signs, patients with post-TBI olfactory dysfunction rarely regain normal olfactory ability
Levin et al. (24)	Short term longitudinal	To investigate the effects of closed head injury on olfactory identification and examine the relevance of TBI severity, location of focal damage on this	Olfaction worse in TBI patients than in non-TBI controls, especially in patients with moderate or severe TBI. Hematoma or contusion in the frontal/temporal regions was related to olfactory recognition
Drummond et al. (25)	Cross sectional	Describe the impact of olfactory impairment on daily activities and social participation from the perspective of the patient with TBI related olfactory impairment	Olfactory dysfunction has significant impact on a range of activities and social roles
Ruff et al. (26)	Longitudinal	Observational study of a cohort of veterans with mild TBI subject to headaches, residual neurological deficits, post-traumatic stress disorder (PTSD). Olfaction was assessed. They were treated with sleep hygiene counseling and prazosin	Reduced clinical manifestations following mTBI correlated with PTSD severity and improvement in sleep, but not olfactory impairment
Joung et al. (27)	Longitudinal	Does frontal skull base fracture have an impact on the occurrence and recovery of anosmia and/or agneusia following frontal TBI	Among 102 patients who had hemorrhage or contusion on the frontal lobes, anosmia was present in 22 (21.6%) of whom 20 had bilateral frontal lobe injuries. Frontal skull base fracture did not otherwise increase the rate of anosmia in this sample but recovery from anosmia was greater in those without fracture
Hirsch and Wyse (28)	Cross sectional	To use an olfactory threshold test and a (suprathreshold) olfactory identification test to infer the localization of the olfactory pathway lesions (i.e., peripheral or central) in 13 patients with post-TBI olfactory impairment	Thirty-eight percent of patients were hyposmic on suprathreshold tests but had normal scores on threshold tests, while 62% had abnormal scores on both threshold and suprathreshold tests. The investigators presume that the former group likely had central causes (possibly cortical) for olfactory impairment, and that they might be more responsive to therapy than the second group with presumptive olfactory nerve dysfunction (perhaps shearing at cribriform plate)

*TBI, traumatic brain injury; mTBI, mild traumatic brain injury; NR, not reported; SPECT, single photon emission computed tomography; CT, cat scan; GCS, Glasgow Coma Scale; PTA, post-traumatic amnesia; LOC, loss of consciousness; AST, Alberta smell test*.

**Table 2:** **Study participant demographics**.

Reference	*N*		TBI sex		Ctrl sex		TBI age		Ctrl age		TBI education		Ctrl education	
	TBI	Ctrl	M	F	M	F	*M*	SD	*M*	SD	*M*	SD	*M*	SD
Parma et al. (4)	12	11	10	2	9	2	37.92	7.56	39.45	8.92	13.83	4.02	15.27	4.1
Neumann et al. (5)	106	0	75	31	N/A	N/A	39.5	Range: 21–66	N/A	N/A	Dys: 12.9	2.6	13.2	2.6
Charland-Verville et al. (6)	22 (SCG = 12; MCG = 10)	13	22	0	13	0	SCG: 22.5; MCG: 23.9	SCG: 1.3; MCG: 2.3	22	2.6	NR	NR	NR	NR
Welge-Lussen et al. (7)	67	0	38	29	N/A	N/A	40.1	Range: 17–66	N/A	N/A	NR	NR	N/A	N/A
Gerami et al. (8)	19	13	10	9	7	6	37.5	±8.0	34.46	±7.12	NR	NR	NR	NR
Sigurdardottir et al. (9)	115	0	Mild: 25; Mod: 25; Sev: 31	Mild: 15 Mod: 9 Sev: 10	N/A	N/A	Mild: 35.9; Mod: 33.5; Sev: 28.5	Mild: 11.4; Mod: 10.8; Sev: 10.4	N/A	N/A	Mild: 14.3; Mod: 12.5; Sev: 12.6	Mild: 2.5; Mod: 3.0; Sev: 1.9	N/A	N/A
Vent et al. (10)	50	0	50	0	NR	0	26.4	9.6 range: 18–57	N/A	N/A	NR	NR	N/A	N/A
Fortin et al. (11)	49	0	36	13	N/A	N/A	42.98	16.41	N/A	N/A	NR	NR	N/A	N/A
Haxel et al. (12)	190	0	146	44	0	0	32.09	12.84	N/A	N/A	NR	MR	N/A	N/A
Rombaux et al. (13)	25	N/A	12	13	N/A	N/A	43.9	Range 20–70	N/A	N/A	NR	NR	N/A	N/A
Sandford et al. (14)	37	36	27	10	NR	NR	10.11	0.47 range: 5–16	10.08	0.48	NR	NR	NR	NR
Green et al. (15)	367	Neuro ctrl: 64; normal ctrl: 196	290	77	Neuro ctrl: 36; norm ctrl: 176	Neuro ctrl: 28; norm ctrl: 20	38.9	12.4	Neuro ctrl: 46.6; norm ctrl: 37.3	Neuro ctrl: 9.4; norm ctrl: 9.5	11.8	2.7	Neuro ctrl: 13.5; norm ctrl: 10.8	Neuro ctrl: 3.7; norm ctrl: 2.2
De Kruijk et al. (16)	111	0	61	50	N/A	N/A	Median: 34	Range: 17–72	N/A	N/A	NR	NR	N/A	N/A
Callahan and Hinkebein (17)	122	0	73%	27%	N/A	N/A	33.13	13.32	N/A	N/A	12.32	1.91	N/A	N/A
Fujii et al. (18)	27	0	13	14	N/A	N/A	38.3	Range: 16–57	N/A	N/A	NR	NR	N/A	N/A
Green and Iverson (19)	322	126	254	68	NR	NR	38.7	12.1	36.9	8.9	11.8	2.7	11	2.3
Yousem et al. (20)	36	24	21	15	12	12	35	11.4	39	11.5	NR	NR	NR	NR
Geisler et al. (21)	25	25	12	13	12	13	45.5	6.7	44.7	4	A = 14.1	A = 0.9	14.1	0.5
											H = 12.7	H = 0.7	
											N = 14.7	N = 0.8	
Callahan and Hinkebein (22)	68	None	51	17	NR	NR	31.97	12.52	NR	NR	11.97	1.8	NR	NR
Doty et al. (23)	268 Sub-group 66	NR	148 Sub-group 35	120 Sub-group 31	NR	NR	M40.4	M16.2	NR	NR	NR	NR	NR	NR
							F42.3	F17.8	
							Sub-group	Sub-group	
							M40.0	M17.9	
							F 50.4	F16.5	
Levin et al. (24)	52	19	NR	NR	NR	NR	Median 20.6	Range 13–42	Median 22.3	Range 18–26	Median 11.8	Range 7–16	Median 13.7	Range 12–21
Drummond et al. (25)	5	NR	4	1	NR	NR	44.8	15.55	NR	NR	NR	NR	NR	NR
Ruff et al. (26)	63	NR	57	6	NR	NR	29.5	SE = 0.92	NR	NR	NR	NR	NR	NR
Joung et al. (27)	22	NR	19	3	NR	NR	44.5	19.15	NR	NR	NR	NR	NR	NR
Hirsch and Wyse (28)	13	0	NR	NR	N/A	N/A	38.46	13.09, range: 19–64	NR	NR	NR	NR	NR	NR

**N*, number; Ctrls, controls; M, male; F, female; SD, standard deviation; Mod, moderate; Sev, Severe; TBI, traumatic brain injury; Ax, assessment; N/A, not applicable; NR, not reported; *M*, mean; SCG, single concussion group; MCG, multiple concussion group; A, anosmic; H, hyposmic; N, normosmic; Norm, normal*.

Traumatic brain injury severity was variously defined by the Glasgow Coma Score (GCS) (5, 6, 9, 15, 16, 19, 24, 25, 27), the duration of post-traumatic amnesia (PTA) (5, 6, 9, 15, 19, 25), the occurrence (and/or duration) of LOC (5, 6, 14, 19, 22, 23) – measures available to the clinician early following TBI or by functional outcomes (e.g., neuropsychological test performance (4, 9, 15, 17, 19, 21, 22, 24, 26), behavioral tests, or other functional questionnaires (5, 6, 9, 10, 22, 26) (Tables 3 and 4). One study focused solely on severe TBI (4), while three were concerned with mild TBI only (6, 10, 16). Structural brain imaging was examined in relation to olfactory outcomes in a number of studies (12–14, 18, 24, 27) (Table 4).

**Table 3:** **TBI characteristics**.

Reference	TBI severity criteria (GCS, PTA, LOC)	Mild TBI (*n*)	Mod TBI (*n*)	Sev TBI (*n*)	GCS score	PTA score	LOC time	Time since injury
Parma et al. (4)	GCS = 3–8	0	0	12	NR	NR	NR	NR
Neumann et al. (5)	GCS (at the time of injury): ≤12; PTA: ≥24 h; LOC: ≥24 h	0	NR	NR	*M:* 5.38	>7 days: 76%	Mean: 53.57 days	*M:* 11.54 years, range 1–42 years
Charland-Verville et al. (6)	Graded from one to three according to the AAN guidelines	22	0	0	All 13–15	*n* = 12; no other details	*n* = 7; no other details	SCG *M:* 26.9 months, SD: 21.9; MCG *M:* 3.9 months, SD: 26.4.
Welge-Lussen et al. (7)	NR	21	24	22	NR	NR	NR	T1: *M:* 16.7 months; T2: *M:* 74 months
Gerami et al. (8)	NR	NR	NR	NR	NR	NR	NR	NR
Sigurdardottir et al. (9)	GCS, PTA, and LOC classification	40	34	41	Mild: *M:* 14.7 (0.6)	Mild: *M:* 0.08 range: 0–1	NR	T1: 3 months; T2: 1 year
					Mod: *M:* 10.8 (1.3)	Mod: *M:* 5.25 range: 0–30	
					Sev: *M:* 5.5 (1.8)	Sev: *M:* 35.83 range: 0–128	
Vent et al. (10)	No TBI as such, rather repetitive blows to the head (boxing).	N/A	N/A	N/A	N/A	N/A	N/A	NR
Fortin et al. (11)	GCS	26	9	14	N/A	N/A	N/A	*M:* 10.49 months (7.30), range 1–40 months, median: 8 months
Haxel et al. (12)	GCS	32	94	64	NR	NR	NR	6–32 months
Rombaux et al. (13)	NR	NR	NR	NR	NR	NR	NR	*M:* 14.9 months, range 3–60 months
Sandford et al. (14)	GCS score	31	3	3	NR	NR	Positive n = 15 (40.5%); Questionable: *n* = 7 (18.9%)	*M:* 189.45, SD: 9.66, range: 113–277 days
Green et al. (15)	GCS: Mild: 13–15; Mod: 9–12; Sev: 3–8	112	12	23	Median Scores: Mld: 15; Mod: 10.5; Sev: 6	Mean (SD) Scores (h): <1 day: 3.0(4.5); 1–10 days: 88.5(55); >10 days: 726(650).	NR	NR
De Kruijk et al. (16)	mTBI defined as PTA <1 h, initial LOC <15 min, GCS 14 or 15 at ED, absence of focal neurological signs	111	0	0	14 or 15	NR	NR	2 weeks post-mTBI
Callahan and Hinkebein (17)	GCS	43	19	60	NR	NR	NR	*M:* 424.30 days, SD: 967.24, range: 17–7854, median: 94.5 days
Fujii et al. (18)	NR	NR	NR	NR	NR	NR	NR	Variable: one group commenced treatment within 2 months and other >2 months
Green and Iverson (19)	NR except for the sev brain injury sub-group GCS <9. Trivial – Mild Injury Group: LOC for <10 min and PTA for <1 h; Definite TBI Group: LOC for >30 min or PTA for >24 h, or an abnormality on brain CT. A subsample of the most sev injured patients was selected from the definite TBI group who met the following: abnormal CT and duration of PTA >72 h, or abnormal CT and GCS <9, or PTA >7 days	137	75	51	Sev TBI <9; NR for other groups	Mild: <1 h; Mod: >24 h; Sev: >72 h OR >7 days	Mild: <10 min; Mod: >30 min; Sev: >30 min	NR
Yousem et al. (20)	NR	NR	NR	NR	NR	NR	NR	Delays between the traumatic event and the MR examination ranged from 3 to 540 months, *M* = 52 (95.6).
Geisler et al. (21)	NR	NR	NR	NR	NR	NR	NR	NR
Callahan and Hinkebein (22)	Admission GCS scores were used to segregate into Mld, Mod and Sev TBI. scores NR	35	12	21	NR	NR	Mean time in coma was 6.04 days (9.55); normosmic group = 2.71 (5.53); Anosmic group = 7.86 (10.78)	281.35 days (696.67) range = 17–5360 days
Doty et al. (23)	NR in main group but in sub-group LOC was used	NR	NR	NR	NR	NR	UPSIT Scores >18 non-anosmic group, *n* = 18; LOC zero *n* = 5; LOC <24 h *n* = 9; LOC >24 h *n* = 4	NR
Levin et al. (24)	GCS, PTA	7	18	27	Mild 13–15 Mod 9–12 Sev <8	NR	NR	Median: Mild 11.1; Mod 7; Sev 3.9
Drummond et al. (25)	GCS, PTA	NR	NR	5	Participant 1–4 10.5 (5.2) Participant 5 UK	25.4(23.5)	NR	363.6(515.7)
Ruff et al. (26)	AOC following the TBI <24 h, LOC <30 min, or PTA <24 h	63	NR	NR	NR	NR	NR	2.5 years
Joung et al. (27)	GCS	NR	NR	NR	13.5	NR	NR	4.5 days
Hirsch and Wyse (28)	NR	NR	NR	NR	NR	NR	NR	NR

**N*, number; Ctrls, controls; M, male; F, female; SD, standard deviation; Mod, moderate; Sev, severe; GCS, Glasgow Coma Scale; PTA, post-traumatic amnesia; LOC, loss of consciousness; TBI, traumatic brain injury; Ax, assessment; N/A, not applicable; NR, not reported; AOC, alteration of consciousness; *M*, mean; AAN, American Academy of Neurology*.

**Table 4:** **Study design characteristics**.

Reference	Study design	Participant inclusion/exclusion criteria	Neuroimaging	Neuropsychological test	Psychological/psychiatric questionnaires or other material	Other types of injury(IES)
Parma et al. (4)	Cross sectional	Inclusion: LCF Scale score >5; normal vision or corrected vision; right handed. Exclusion: aphasia, apraxia, ataxia, drug abuse, previous neurological disease	N/A	RPM; TMT; verbal span	BAI; BDI; Edinburgh Handedness Inventory; Questionnaire previous Hx nasal disease, smoking Hx current subjective status of olfactory function	NR
Neumann et al. (5)	Cross sectional	Inclusion: moderate to severe TBI determined either by GCS at the time of injury (≤12), PTA ≥24 h, LOC ≥24 h; 18–65-years-old, minimum 1 year post-injury; sufficient comprehension on the DCT. Exclusion: TBI occurred prior to 8 years of age; premorbid developmental or acquired neurological disorder; premorbid major psychiatric disorder; impaired vision and/or hearing; current substance abuse	N/A	N/A	Diagnostic Ax of non-verbal affect 2 – adult faces and paralanguage; emotional interference from Stories Test; interpersonal reactivity index	NR
Charland-Verville et al. (6)	Cross sectional	Inclusion: active players of a uni football team, ≥18-years-old, no Hx of alcohol and/or substance abuse, no medical condition requiring daily medication or radiotherapy, no nasal surgery, smoking, allergies, or common cold symptoms at the moment of testing, no previous Hx of psychiatric illness, learning disability, neurological Hx, or TBI unrelated to contact sport. Exclusion: suffered their last concussion previous to 16-years-old	N/A	N/A	Brief questionnaire concerning general health; semi-structured interview – Hx of concussion/TBI	NR
Welge-Lussen et al. (7)	Longitudinal	NR	NR	N/A	N/A	NR
Gerami et al. (8)	Cross sectional	Exclusion: neurological or systemic disease, with rhinologic or skull base surgeries, with severe septal deviation and nasal masses; consumed vasoactive drugs or alcohol or cigarettes	SPECT	N/A	N/A	NR
Sigurdardottir et al. (9)	Longitudinal	Inclusion: presence of LOC or PTA, skull fracture, or objective neurological findings. GCS used to measure the level of TBI severity. Aged 16–55 years; admission to hospital <24 h post-injury; CT scan performed within 24 h of injury; fluent Norwegian speakers. Exclusion: severe substance abuse; known psychiatric or brain pathology; associated SCI	At 1 year only: MRI	At 3 months only: Iowa Gambling Task; D-KEFS: Verbal Fluency, Design Fluency, Color-Word Interference	The Galveston Orientation and Amnesia Test; The CAGE – screen for premorbid D&A issues	NR
Vent et al. (10)	Cross sectional	Inclusion: healthy males aged 18 years and older. Exclusion: females, previous nasal surgery, chronic rhinosinusitis, nasal polyps, allergies, and medication affecting the olfactory system	N/A	N/A	Standardized boxing Hx questionnaire	N/A
Fortin et al. (11)	Cross sectional	Exclusion: past repeated exposure to vaporous chemicals, consumption of inhaled non-medical drugs, rhinosinusitis, past TBI	N/A	N/A	BDIS	N/A
Haxel et al. (12)	Cross sectional	NR	Radiological exam	N/A	N/A	Fractured cheekbone *n* = 93 (48.9%); fracture of the vertex *n* = 17 (9%); base of the skull fracture *n* = 48 (25%); radiologically verified intracranial injuries *n* = 32 (17%)
Rombaux et al. (13)	Cross sectional	NR	MRI: OB vol; right OB vol, (mm^3^): *M*: 19.2, SD: 9.1 left OB vol. (mm^3^): *M*: 17.6, SD: 9.7; L + R OB vol. (mm^3^): *M*: 36.9, SD: 18.3	N/A	N/A	NR
Sandford et al. (14)	Cross sectional	Exclusion: Hx of DD, craniofacial abnormalities, prior hospitalization for HI, intracranial surgery, chronic illness, dependent mouth breathing, no documented Hx of TBI	CT brain findings: parietal fracture (*N* = 1); maxillary fracture (*n* = 1) and subarachnoid and intraventricular hemorrhages, occipital fracture (*n* = 1), and epidural hemorrhage, temporal fracture (*n* = 1)	N/A	N/A	CT findings: parietal fracture (*N* = 1); maxillary fracture (*n* = 1), occipital fracture (*n* = 1), temporal fracture (*n* = 1)
Green et al. (15)	Cross sectional	Consecutive referrals to a private practice in Edmonton, Alberta, Canada for psychological or neuropsychological assessment, with a TBI or a neurological disease	CT/MRI	WCST; Verbal Fluency; Figural Fluency; Gorham’s Proverbs; CVLT; WRMT–Words and Faces; Cognisyst Story Recall Test; CARB; WMT–Paired Associates, Multiple Choice; RCFT; TMT; Digit Span; Visual Memory Span; WRAT-III–Reading	N/A	NR
				Spelling and Arithmetic; Benton’s JLO; Finger Tapping, Grip strength and Grooved Pegboard	
De Kruijk et al. (16)	Cross sectional	Inclusion: first time mTBI, ≥16 years age, presentation to the ED within 6 h of TBI. Exclusion: suffered from multiple trauma or need for clinical obs, Hx of TBI, alcohol abuse, or psych disorder	N/A	N/A	S-100B, NSE	N/A
Callahan and Hinkebein (17)	Cross sectional	Exclusion: ongoing PTA, presence of severe aphasia, too acute for valid assessment	N/A	NP Ax battery: Mean FSIQ: 88.53, SD: 12.6	N/A	N/A
Fujii et al. (18)	Longitudinal	NR	MRI (intracranial damage) 17/27 cases; anterior skull base (i.e., brain hemorrhage): 7 No findings: 10	N/A	N/A	NR
Green and Iverson (19)	Cross sectional	Inclusion: referred to a private practice in Edmonton, Alberta, Canada; TBI inclusion: (a) Trivial – Mild Injury Group: LOC <10 min and PTA <1 h; and (b) Definite TBI Group: LOC >30 min or PTA >24 h, or an abnormality on brain CT. A subsample of the most severely injured patients was selected from the definite TBI group who met the following criteria: (a) abnormal CT and duration of PTA >72 h, or (b) abnormal CT and GCS <9, or (c) PTA >7 days	CT	CARB and WMT, others NR	NR	NR
Yousem et al. (20)	Cross sectional	Inclusion: Hx of head trauma, referred to the University of Pennsylvania Smell and Taste Center, signed consent, passing scores on the PIT and MMSE. Exclusion: patients with causes or complaints of olfactory dysfunction prior to the date of trauma	MRI	PIT; MMSE	NR	NR
Geisler et al. (21)	Cross sectional	Exclusion: mechanical damage to nasal passage, nasal polyps, deviated septums	NR	TMT	NR	NR
Callahan and Hinkebein (22)	Cross sectional	Inclusion: consecutive admissions to the inpatient and outpatient brain injury rehabilitation programs of a Midwestern medical center must have had a TBI. Exclusion: ongoing state of posttraumatic amnesia, presence of severe aphasia, or being too acute for valid Ax	NR	WAIS-R Digit span, digit symbol; similarities; CVLT-V, WCST, COWAT	Community Integration Questionnaire – Productive Activity	NR
Doty et al. (23)	Cross sectional sub-group longitudinal	Inclusion: previous TBI, sub-group Inclusion: MRI capable	MRI	NR	BDI	NR
Levin et al. (24)	Short term longitudinal	Inclusion: No Hx of neuropsychiatric disorders prior to the head injury; no previous head injury or Hx of alcohol or drug abuse	CT or surgical findings	Orientation and amnesia; visual discrimination; visual naming; auditory comprehension	NR	NR
		Exclusion: ≤50 years, in view of the decline in olfactory performance in older adults	
Drummond et al. (25)	Cross sectional	Inclusion: >18 years; emerged from PTA at least 1 month prior to participation, have sufficient communication and cognitive skills to support the interview process, have no reported or documented nasal surgery or olfactory disturbance pre-injury and have no significant past psychiatric Hx Exclusion: Significant past psychiatric Hx.	CT, MRI	NR	N/A	(1) CHI; (2) CHI, # (L) squamous/temporal bone, scalp/nasal lacerations; (3) CHI, 2 facial lacerations, # (R) and (L) maxilla, # (L) zygoma, CSF leak (L) ear; (4) CHI, undisplaced (R) lateral orbital wall and zygomatic arch, fractured left pterygoid plate, moderate right pre-septal hematoma (globe intact)
Ruff et al. (26)	Longitudinal	Inclusion: mTBI associated with an explosion in operation Iraqi Freedom/Operation Enduring Freedom veterans	NR	MOCA	PCL-M, ESS, DVA TBI screening scale	NR
Joung et al. (27)	Longitudinal	Inclusion: all patients had radiographic evidences of contusion or hemorrhage on the frontal lobe base. These patients were selected on the basis of the neurosurgical diagnosis as a main diagnostic code at the time of hospital discharge, ICD-10	CT	NR	NR	NR
Hirsch and Wyse (28)	Cross sectional	NR	Comprehensive neurologic evaluation, electrophysiological tests; electroencephalogram FFT analysis, P3000 cognitive auditory evoked response, BAER, and VER	N/A	MMPI, MCMI, BDI, blood tests: vitamin B12, RBC folate level, FTA, ESR, CBC, glucose, liver function, electrolytes, total eosinophilic count, IgE level, PT, PTT, and platelet count. Urinalysis and 24 h urinary MHPG levels	NR

**N*, number; Ctrls, controls; M, male; F, female; SD, standard deviation; TBI, traumatic brain injury; CHI, closed head injury; #, fractured; (R), right; (L), left; Hx, history; NP, neuropsychological; Ax, assessment; LFC, level of cognitive functioning; TMT, trail making test; WMC, white matter change; ICD-10, international disease classification-10; D&A, drug and alcohol; N/A, not applicable; NR, not reported; obs, observation; SPECT, single photon emission computed tomography; CAGE, cut down, annoyed, guilty, eye opener; MMSE, mini mental status examination; PIT, picture identification test; CT, discourse comprehension test; BDI, beck depression inventory; BAI, beck anxiety inventory; JLO, judgment of line orientation; PCL-M, PTSD checklist-military version; ESS, Epworth sleepiness scale; COWAT, controlled oral word association test; CVLT, California verbal learning test; RPM, Ravens progressive matrices; CARB, computerized assessment of response bias; WMT, word memory test; WCST, Wisconsin card sorting test; WRMT, Warrington recognition memory test; MOCA, Montreal cognitive assessment; FFT, fast Fourier transform; BAER, brain stem auditory evoked response; VER, visual evoked response; MMPI, Minnesota multiphasic personality inventory; MCMI, Millon clinical multiaxial inventory; FTA, fluorescent treponemal antibody; ESR, erythrocyte sedimentation rate; CBC, complete blood count; PT, prothrombin time; PTT, partial thromboplastin time; MHPG, 3 methoxy-4-hydroxyphenylglycol; LOC, loss of consciousness; PTA, post-traumatic amnesia; DVA, Department Of Veteran Affairs; RBC, red blood cells; NSE, biochemical serum marker; Vol, volume; D-KEFS, Delis–Kaplan executive function system*.

In total, the studies referenced at least three olfactory constructs (sensitivity/threshold, identification, discrimination), 13 olfactory “instruments” [including olfactory event related potentials (ERPs)], administered in at least three different ways (unirhinal, birhinal, retronasal) and cited olfactory outcomes classified either categorically (e.g., normal/hyposmia/anosmia), as raw or scaled scores (relative to population norms) or according to change in olfactory performance over time. Olfactory “impairment” was, for the most part, defined by reference to a defined threshold or cut-off on an olfactory test. This approach also provided the opportunity to report a rate (incidence or prevalence) of impairment. In other studies, a significant mean reduction in olfactory performance in the post-TBI group relative to appropriate controls was reported, indicating impairment in some individuals. The University of Pennsylvania Smell Identification Test (UPSIT) was the olfactory instrument most commonly used (4, 8, 11, 17, 20–23, 28) followed by the Sniffin’ Sticks (6, 7, 10, 12) (Tables 5–8).

**Table 5:** **UPSIT studies**.

Reference	Definition of olfactory deficits (i.e., Anosmia if …)	Unirhinal or birhinal	Smell testing results
**SEVERE TBI**			
Parma et al. (4)	NR	NR	aTBI: *M* = 21.17, SD = 8.65; Ctrl: *M* = 37.27, SD = 3.77
**MULTIPLE LEVELS OF TBI SEVERITY**			
Callahan and Hinkebein (17)	Partial anosmia: <10th percentile on the UPSIT Anosmia: <first percentile	NR	Impaired olfaction: mTBI: 44.2%, moderate TBI: 68.4%, severe TBI: 61.0%
			Awareness of olfactory deficits: mTBI: 23.3% unaware of deficit, moderate TBI: 47.4%, severe TBI: 48.3%
Callahan and Hinkebein (22)	Partial anosmia <10th percentile on UPSIT; total anosmia <first percentile on UPSIT. No significant differences were found between the partial and total anosmia groups on any variable, so were combined into one anosmic group (*N* = 44) for further analyses	NR	Forty-four participants (65% of total sample) demonstrated impaired olfaction: 30 with partial anosmia (<tenth percentile on the UPSIT) and 14 with total anosmia (<first percentile)
			The normosmic group (normal smell function) consisted of 24 participants. UPSIT scores did not correlate with any other dependent variable. Interestingly, 31 of these 44 anosmic participants (70%) denied any smell or taste problems in clinical interview
**SEVERITY NOT REPORTED**			
Gerami et al. (8)	N/A	NR	TBI *M*: 11.2, SD: 2.7
			Ctrl *M*: 36.7, SD: 3.2 significant diff
Yousem et al. (20)	Anosmia = UPSIT score <18	Unirhinal	UPSIT total: All TBI 21.9 (10.5); anosmia 10.5 (3.0); hyposmia 28.4 (6.9); ctrl 36.6(2.6)
	Hyposmia = UPSIT score >18		UPSIT left: all TBI 10.5(5.2); anosmia 5.2(2); hyposmia 13.5 (3.9); Ctrl 18.5(1.6)
			UPSIT right: All TBI 11.4 (5.6); anosmia 5.2 (1.8); hyposmia 14.9(3.5); Ctrl 18.1(1.7)
			Odor Memory Scores, left: all TBI 5.3(3.1); anosmia 2.7(0.95); hyposmia 6.8(2.9); Ctrl 9(2.7)
			Odor Memory Scores, right: all TBI 5.1 (3); anosmia 3.3 (1.7); hyposmia 6.2 (3.2); Ctrl 9.4 (1.8)
			Thresholds, left: all TBI −4 (2.9); anosmia −2 (0.51); hyposmia −5.1 (3.1); Ctrl −6.9(2.3)
			Thresholds, right: all TBI −3.9 (2.7); anosmia −1.9 (0); hyposmia −5.1 (2.8); ctrl −7 (2.1)
Geisler et al. (21)	NR	Birhinal	UPSIT: anosmic 10.3 (1); hyposmic 21.2 (3.1); normosmic 35.4 (1.3); ctrl 37.7 (0.9)
			Amyl acetate threshold: anosmic 0.3(0.3); hyposmic 2.1(0.7); normosmic 6.5(0.7); ctrl 8.5(0.2)
			Butanol threshold: anosmic 0.4 (0.2); hyposmic 3.6 (0.8); normosmic 5.3 (0.2); ctrl 8 (0.3)
			Odor identification: anosmic 0.1 (0.1); hyposmic 3.5 (0.9); normosmic 6.6 (0.4); ctrl 7.6 (0.2)
			Alcohol Sniff Test: anosmic 1.1 (0.6); hyposmic 16.8 (5); normosmic 22.1 (3.8); ctrl 28.3 (0.5)
Doty et al. (23)	Anosmia – total inability to smell (UPSIT scores >5 and <19)	NR	One hundred seventy-nine patients (66.8%) had anosmia, 55 (20.5%) had microsmia, and 34 (12.7%) had normosmia
	Microsmia – lessened ability to smell (men: UPSIT scores ranging from 19 to 33; women: UPSIT 19–34)		Frontal impacts produced less dysfunction than back or side impacts
	Normosmia – no meaningful olfactory loss (men: UPSIT ~34; Women: UPSIT ~35). microsmia – mild microsmia (Men: UPSIT 30–33; Women: UPSIT 31–34)		Of the 66 retested patients, 24 (36%) improved slightly, 30 (45%) had no change, and 12 (18%) worsened; only 3 patients, none of whom initially had anosmia, regained normal olfactory function
	Moderate microsmia (Men: UPSIT 26–29; Women: UPSIT 26–30)		Trauma severity was related to olfactory test scores in patients with microsmia
	Severe microsmia (UPSIT 19–25)		

**N*, number; Ctrls, controls; *M*, mean; SD, standard deviation; UPSIT, University of Pennsylvania Smell Identification Test; N/A, not applicable; NR, not reported; ODT, odor discrimination Test; OMT, odor memory test; TBI: traumatic brain injury; aTBI: anosmic TBI*.

**Table 6:** **Sniffin’ sticks studies**.

Reference	Definition of olfactory deficits (i.e., anosmia if …)	Unirhinal or birhinal	Smell testing results
**MILD TBI**			
Charland-Verville et al. (6)	NR	NR	SCG: threshold *M*: 9.50, SD: 3.43; discrimination *M*: 22.69, SD: 4.35; ID *M*: 13.77, SD: 1.42; total score *M*: 46.04, SD: 5.70; Ave intensity rating *M*: 73.48 SD: 14.66; Ave pleasantness rating *M*: 59.73, SD: 14.57
			MCG: threshold *M*: 9.50, SD: 4.15; discrimination *M*: 23.30, SD: 4.22; ID *M*: 14.10, SD: 1.52; total score *M*: 46.90, SD: 7.23; Ave intensity rating *M*: 69.89 SD: 10.32; Ave pleasantness rating *M*: 65.43, SD: 14.52. No sig diffs observed
			(33 months: threshold *M*: 10.25, SD: 3.31; discrimination *M*: 24.18, SD: 3.66; ID *M*: 14.80, SD: 1.14; total score *M*: 49.43, SD: 5.42; Ave intensity rating *M*: 75.06 SD: 12.58; Ave pleasantness rating *M*: 64.80, SD: 11.31
			>33 months: threshold *M*: 8.30, SD: 3.88; discrimination *M*: 21.18, SD: 4.19; ID *M*: 13.0, SD: 1.27; total score *M*: 42.48, SD: 5.02; Ave intensity rating *M*: 70.13 SD: 13.84; Ave pleasantness rating *M*: 63.97, SD: 15.23
			Sig diffs ID and Total score 0.05 level (0.003 and 0.007) and Discrim at 0.01 level (0.09)
**MULTIPLE LEVELS OF TBI SEVERITY**			
Welge-Lussen et al. (7)	TDI score (range 1–48); functional anosmia: ≤15; hyposmia 15–30; normosmia >30. A significant side difference was defined as ≥6 point difference on the TDI between nostrils	Birhinal	TDI (right): *M*: T1 = 12.76 vs. T2 = 16.01; significant improvement
			TDI (left): *M*: T1 = 13.58 vs. T2 = 16.35; significant improvement
			Mean for each subtest also improved significantly over T1 and T2. According to the results of the best nostril – T1 classification = anosmia 37 (55.2%); hyposmia 27 (40.2%); normosmia 3 (4.5%)
			T2 classification = anosmia 25 (37.3%); hyposmia 35 (52.2%); normosmia 7 (10.4%)
			In 18 (27%) TDI improved >6 points; 3 (4.5%) TDI decline >6 points
			TBI severity was not a significant influence on the results
**SEVERITY NOT REPORTED**			
Vent et al. (10)	TDI score	NR	Hyposmic: 14 mean TDI: 27.8, SD: 2.1
			Normosmic: 36 mean TDI: 34.3, SD: 2.4

**N*, number; Ctrls, controls; *M*, mean; SD, standard deviation; UPSIT, University of Pennsylvania Smell Identification Test; N/A, not applicable; NR, not reported; sig diffs, significant difference; SCG, single concussion group; MCG, multiple concussion group; TDI, threshold, discrimination and identification*.

**Table 7:** **Other olfactory tests studies**.

Reference	Definition of olfactory deficits (i.e., anosmia if …)	Unirhinal or birhinal	Smell testing results
**BRIEF SMELL IDENTIFICATION TEST (B-SIT)**			
**Mild TBI**
Neumann et al. (5)	NR	Birhinal	No specific B-SIT results were reported
Ruff et al. (26)	NR	NR	Olfaction Score [Mean (SE)] – baseline 4.13 ± 0.11; Δ (baseline–9 weeks) 0.0925 ± 0.18; Δ (baseline–final) 0.0635 ± 0.15; Δ(9 weeks–final) 0.0318 ± 0.14
**Multiple levels of TBI severity**
Sigurdardottir et al. (9)	Olfactory dysfunction: B-SIT score of <9 anosmic: unable to Id any smells on the B-SIT	NR	At 3 months: 33 (30%) olfactory dysfunction. mild TBI (score <9) at 3 months: 8 (20%); moderate TBI (score <9) at 3 months: 12 (37%); severe TBI (score <9) at 3 months: 13 (33%). Of the 33 with deficits at 3 months, recovery at 12 months was observed in 13 (39%). Mild TBI (score <9) at 1 year: 6 (15%); moderate TBI (score <9) at 1 year: 5 (15%); severe TBI (score <9) at 1 year: 9 (22%); anosmic: severe TBI: 10%
**ALBERTA SMELL TEST**			
**Multiple levels of TBI severity**
Green et al. (15)	Could not smell anything at all	Unirhinal	Mean (SD)
			Mild: 5.18 (2.6); moderate: 2.92 (2.9); severe: 3.41 (2.7)
Green and Iverson (19)	NR	Unirhinal	Mean (SD): right nostril-trivial TBI adequate effort (*n* = 86): 5.8 (2.2); definite TBI adequate effort (*n* = 93): 3.6 (2.9); Severe TBI adequate effort (*n* = 40): 3.7 (3.1); trivial TBI poor effort (*n* = 51): 4.7 (2.8). Left nostril-trivial TBI adequate effort (*n* = 86): 6.2 (2.2); Definite TBI adequate effort (*n* = 93): 3.7 (3.0); Severe TBI adequate effort (*n* = 40): 3.4 (3.2); trivial TBI poor effort (*n* = 51): 4.9 (2.7). Total nostril-trivial TBI adequate effort (*n* = 86): 12.0 (3.9); definite TBI adequate effort (*n* = 93): 7.3 (5.4); severe TBI adequate effort (*n* = 40): 7.0 (5.9); trivial TBI Poor effort (*n* = 51): 9.6 (4.9). Patients with severe TBI were 12.0 times more likely to demonstrate impaired olfaction than the patients with very mild injuries
**HYPOSMIA UTILITY KIT**			
**Mild TBI**
De Kruijk et al. (16)	Hyposmia: threshold range 30–55dS; anosmia: nil detection of odor at 55dS	NR	Of the 111 in sample, one-quarter revealed quantitative olfactory dysfunction after 2 weeks, while 22% had hyposmia and 4% had anosmia
**OLFACTORY IDENTIFICATION TEST**			
**Multiple levels of TBI severity**
Levin et al. (24)	NR	Birhinal	Medians: Olfactory naming (0–12); mild 3.3; moderate 2; severe 0.7; Ctrl 4.1; olfactory recognition (0–12): mild 9.7; moderate 8.3; severe 8.1; Ctrl 9.9
**T&T OLFACTOMETER**			
Joung et al. (27)	Scored 0/2 on olfaction test	NR	Anosmia – frontal skull base fracture present *n* = 9 frontal skull base fracture absent *n* = 13. No statistical difference
			However, when recovery from anosmia was considered, six patients without fracture showed recovery (66.7%) while only three patients with fracture showed such recovery (23.1%) (*p* < 0.05)

**N*, number; Ctrls, controls; *M*, mean; SD, standard deviation; UPSIT, University of Pennsylvania Smell Identification Test; N/A, not applicable; NR, not reported; dS, olfactory threshold, B-SIT, brief smell Id test; Δ, change*.

**Table 8:** **Multiple olfactory test studies**.

Reference	Olfactory tests used	Definition of olfactory deficits (i.e., anosmia if …)	Unirhinal or birhinal	Smell testing results
**SEVERE TBI**				
Drummond et al. (25)	UPSIT	NR	Birhinal	Three males got 0/3 on PST and testing ceased
	PST			One female and one male got 3/3 and 2/3 respectively then proceeded to complete the UPSIT and got 30/40 and 15/40, respectively
**MULTIPLE LEVELS OF TBI SEVERITY**				
Fortin et al. (11)	UPSIT AST	N/A	NR	UPSIT: 39 (69%) demonstrated impaired olfaction; anosmia: 11, hyposmia 28; 44% (*n* = 17) were unaware of their deficit. AST: 27 (55%) demonstrated impaired olfaction; 41% (*n* = 11) were unaware of a deficit. The Dx of both tests was in agreement for 62% of the cases; sig +ve corr. (*r* = 0.82, *p* < 0.05)
Haxel et al. (12)	Brief Smell Id Test (B-SIT) (*n* = 82) SS (*n* = 19) Chemosensory event-related potentials	N/A	Birhinal	*n* = 21 (11%) reported reduced sense of smell; B-SIT: *n* = 82 completed (60 males, 22 females; *M* age 31, SD: 13.1, 37 smokers) scored ≤8. SS: *n* = 19 (16 self-reported olfactory impairment, three scored ≤8 on B-SIT) completed (17 males, 2 females; 10 smokers, 3 ex-smokers; age ranged 21–57, *M*: 37, SD: 11.5; *n* = 9 TDI score within normal limits (28, 25–34); *n* = 3 hyposmic (TDI: 18–26.5); *n* = 7 anosmic (TDI: 10–15.5). B-SIT was found to have a sensitivity of 100% and a specificity of 33% (when the SS are taken as the “gold standard”). Corr. between the olfactory function test was 0.73 (*P* < 0.05). The overall incidence of olfactory dysfunction was estimated to be 12.8%
Sandford et al. (14)	San Diego odor identification test	NR	NR	Odor identification % age score: TBI – *M*: 90.88, SD: 2.00; Ctrl – *M*: 94.52, SD: 2.03. Of the 36 TBI patients; 3 failed the odor ID task (defined as 6 of 8 incorrect odors = hyposmic)
	Olfactory event-related potentials	
**SEVERITY NOT REPORTED**				
Rombaux et al. (13)	SS Retronasal testing	Diagnostic criteria for posttraumatic olfactory loss: (1) A Hx of olfactory disorder after TBI, (2) patency of the olfactory cleft at endoscopic exam, (3) evidence of olfactory dysfunction, (4) exclusion of other causes of olfactory disorders. Parosmia was defined as the perception of distorted odors in the presence of an odor source	Birhinal	SS: detected 20 patients as anosomia; 5 hyposmic. TDI scores *M*: 12, SD: 5.8 (range 3–29); odor threshold *M*: 2.52, SD: 1,5; odor discrimination *M*: 4.96, SD: 2.3; odor ID *M*: 4.64, SD: 2.9 Retronasal Testing: *M*: 9.6, SD: 3.1
Fujii et al. (18)	T&T Olfactometry Alinamin test	T&T Olfactometry group classifications: anosmia: >5.5; severe hyposmia: 4.1–5.5 Moderate hyposmia: 2.6–4.0 Mild hyposmia: 1.1–2.5	NR	Anosmia: 16 (61.5%); severe hyposmia: 5 (19.2%); moderate hyposmia: 3 (11.5%); mild hyposmia: 2 (7.7%); alinamin test: 8 cases were positive (30.8%); 18 cases (69.2%) were negative. In the MRI (abnormal findings group, *n* = 7); anosmia: 6 (85.7%), moderate microsmia: 1 (14.3%); normal MRI, *n* = 10); anosmia 5 (50%); severe microsmia 3 (30%); moderate microsmia 1 (10%), mild microsmia 1 (10%)
Hirsch and Wyse (28)	UPSIT	Hyposmic: ≤35/40 on UPSIT, ≤ 6/20 on the CHOT, threshold olfactory test levels >30dS. anosmic: threshold olfactory test levels >55dS	Unirhinal	38% had normal thresholds for odor detection but impaired ability to identify suprathreshold concentrations of odorants; 62% had abnormal thresholds for both the threshold and suprathreshold tests
	CHOT (*n* = 12)	
	Threshold olfactory test	

**N*, number; Ctrls, controls; *M*, mean; SD, standard deviation; AST, Alberta smell test; SS, Sniffin’ Sticks; CHOT, Connecticut Home Olfactory Test; UPSIT, University of Pennsylvania Smell Identification Test; PST, pocket smell test; N/A, not applicable; NR, not reported; sig, significant; +ve, positive; Corr, correlation; % age, percentage; dS, olfactory threshold; Dx, diagnosis*.

The interval between the TBI and olfactory testing ranged from 2 weeks (16) to many years (5, 17), and in some cases, it was not reported. Several studies compared different techniques for assessing post-TBI olfaction, including functional brain imaging or electrophysiological techniques (8, 12, 21). A single study employed qualitative techniques to focus on the functional consequences of post-TBI olfactory impairment (25) (Tables 5–8).

### Main findings

Of the studies that examined olfaction following mild TBI (6, 16) [or presumptive mild TBI (10)], the findings were mixed. Among athletes reporting concussion, there were no differences on olfactory testing relative to controls although, surprisingly, longer elapsed time since the most recent concussion was associated with significantly worse olfaction (6). This finding contrasted with those from longitudinal studies based on individuals with more severe TBI in which the overall trend was toward improvement in olfactory test performance over time, although post-TBI anosmia rarely if ever reverted to normal olfaction (7, 23). Among 111 individuals with mild TBI, 26% scored in an impaired range on an olfactory threshold test at 2 weeks post-injury (16). In a study of boxers including, apparently, many currently active in the sport, of whom approximately one-third had experienced at least one “knock out,” 28% were hyposmic, and as a group their olfactory performance was significantly worse than their matched controls (10). The results suggest that mild TBI or recurrent blows to the head might have an impact on olfaction, at least in the short term.

Of the studies that examined the relationship between olfactory function and severity of TBI, the findings were also mixed although for the most part they indicated an association (11, 14, 15, 17, 19, 24). A methodologically strong study by Levin et al. (24) included controls and the sample selection minimized bias; three different means of defining TBI severity (GCS, PTA, duration of LOC) were employed; and olfactory functioning was assessed using the Olfactory Identification Test (which includes naming and recognition trials). Olfactory tests were administered between 0.2 and 84 months after the injury and in all cases after resolution of PTA. Individuals with a GCS within the 13–15 range with mass lesions were combined with data from the GCS 9–12 group. Based on this assignment of severity, both the moderate and severe groups differed from the control group on olfactory tests and the trend for a decline in olfactory naming and recognition from mild to moderate and severe groups approached significance. Similar findings held when the duration of impaired consciousness or the duration of PTA was used to classify TBI severity. In each case, the mild group did not differ significantly from controls. Using the UPSIT, Callahan and Hinkebein found an inverse association with injury severity (GCS), but the relationship only emerged with the exclusion of individuals who failed tests of effort, many of whom had mild TBI and did relatively more poorly on olfactory testing (17). Sandford et al. divided pediatric patients with TBI into severity groups according to GCS, and assessed olfaction using the San Diego Children’s Odor Identification test (14). Only three children had olfactory dysfunction and as a group they had lower GCSs than those without. In another study, of 115 individuals examined at 3 months and 1 year post-TBI, the incidence of olfactory dysfunction was 22.3 and 13.5%, respectively [based on the Brief Smell Identification Test (B-SIT)] and there was no relationship of olfaction with TBI severity levels as defined by GCS (9). However, anosmia was more common in the severe TBI group, and performance on the B-SIT was significantly associated with verbal fluency performance, arguably a proxy for injury severity. A similar correspondence of olfactory test results with neuropsychological test performance was also evident in the studies of Green and colleagues (15, 19). Fortin et al. found that 69% of 49 individuals admitted to an outpatient’s rehabilitation program demonstrated impaired olfaction with no difference in rates according to TBI severity (11).

Estimates of the risk of olfactory dysfunction following a TBI are likely to vary in part due to factors, such as study-specific differences in the choice of olfactory tests and the cut-offs for “impairment,” different spectrums of TBI severity (and the means by which this was determined), and the interval of time since injury. Within the clinical studies reviewed in detail, the reported prevalence of olfactory dysfunction among cases with “mild” TBI was: 20% (9), 23% (15), 26% (16), 44% (17); among those with “moderate” TBI: 37% (9), 68.4% (17); with “moderate to severe” TBI: 49% (15), 56% (5); and with severe TBI:33% (9), 61% (17).

Haxel et al. attempted to minimize bias by using a stepped approach to the detection of olfactory dysfunction in a TBI-injured group identified through hospital records (12). Using a combination of subjective report, screening with the B-SIT, and definitive testing with the Sniffin’ Sticks, they estimated an overall prevalence of post-TBI olfactory dysfunction of 12.8% (12). In agreement with several other studies, they found significant rates of unawareness of olfactory dysfunction (5, 11, 17, 22) and evidence for increased risk of olfactory impairment associated with fractures to the base of skull and/or frontal hematomas (15, 20, 23, 24, 27). Hirsch and Wyse administered suprathreshold olfactory identification tests (the UPSIT and the Connecticut Home Olfactory Test) and olfactory threshold tests and defined two patterns of post-TBI dysomia (28). In one group, olfactory sensitivity was impaired but odor identification preserved. In the second group, scores were abnormal on both tests. They hypothesized that the first pattern would be consistent with peripheral pathology (e.g., olfactory nerves) while the second pattern might reflect pathology in central olfactory pathways or centers (28). Yousem et al. obtained high resolution magnetic resonance images of the olfactory bulbs and tracts and temporal lobes and related the findings to performance on the UPSIT, an odor memory test, and an olfactory threshold test, in cases with TBI and controls (20). The olfactory bulbs and tracts (89%), subfrontal lobes (61%), and temporal lobes (31%) of 36 patients showed the highest incidence of encephalomalacia and the left olfactory bulb and tract volumes showed a significant correlation with left and total UPSIT scores (20). The close proximity of the olfactory bulbs and tracts to the frontal lobes made dual pathology commonplace.

Several studies sought associations between post-TBI olfactory dysfunction and behavioral measures or other markers of neuropsychiatric dysfunction (5, 9, 22). In one study, individuals with post-TBI dysosmia performed more poorly on tests of affect recognition, emotional inference, and empathy (5). In another study, despite comparable GCS scores, the anosmic group displayed worse performance than the normosmic group on tests of memory and executive functioning and greater functional impairment as coded by the Disability Rating Scale (22).

Drummond, Douglas, and Oliver administered a semi-structured interview to five individuals who had sustained a severe TBI and had demonstrated olfactory dysfunction. All participants reported that the olfactory impairment had limited their ability to engage in specific activities including eating and enjoyment of food, food preparation, personal safety and hygiene, work, leisure, and personal relationships (25).

## Discussion

The potential consequences of TBI are diverse. Injury severity varies enormously and numerous, complex pathophysiological mechanisms initiated by TBI alter the brain function acutely and beyond. Neuroanatomical and kinetic factors render the peripheral and central olfactory structures highly vulnerable to TBI-related damage, as reflected in the high prevalence of post-injury olfactory dysfunction reported here.

The results of this review confirm that post-TBI olfactory dysfunction is common. If persistent, it represents the loss of an important sensory function with potential functional consequences as eloquently outlined in the qualitative study cited immediately above (25). Remarkably, many individuals who suffer this complication appear to be unaware of it. Finally, its presence seems to signal an increased likelihood of adverse cognitive and other neuropsychiatric and functional outcomes. The implications of these findings are worth considering at the extremes of TBI severity.

Individuals who sustain *severe* TBI typically undergo brain imaging and receive medical services and rehabilitative efforts over extended periods. Optimally, neuropsychiatric and neuropsychological assessments are a routine component of this care. Ideally, this comprehensive and extended engagement should explicitly identify all TBI-related complications. The cognitive and behavioral changes of severe TBI are likely to diminish the capacity to deal effectively with risky situations such as, for example, fire or a gas leak. Identification before discharge of profound anosmia, which would further impair the early detection of such dangers could be justified as a clinical imperative. Severe TBI may lead to changes in social and occupational roles including, perhaps in males particularly, an increased role with food preparation with inherent risks for individuals with anosmia.

By contrast, “mild” TBI often attracts minimal if any clinical evaluation. Nevertheless, in some cases it may be far from benign. Intracerebral contusion and/or hemorrhage, as well as persisting cognitive or behavioral changes have been reported, albeit uncommonly (24). Several of the studies included in this review suggest that the identification of dysosmia/anosmia in a patient with mild TBI could serve to flag an increased likelihood of such unexpected complications (22, 24). In our view, more high quality studies evaluating olfaction and its correlates following mild TBI are needed. In light of the limited scope of the existing studies in this patient group, clarification of the evolution and clinical significance of any early post-injury olfactory changes would be invaluable.

The number and diversity of olfactory instruments and techniques available present a challenge for clinicians and researchers who wish to compare results across studies. It is beyond the scope of this review to venture specific recommendations regarding the “best” choice of instruments or the design of a “minimum data set” for future studies, even if the goal of “harmonization” is a worthy one. Financial resources, time, context, and the particular research question at hand clearly bring their own imperatives and necessarily influence such choices. As a general principle, however, the use of olfactory tests with good normative data, appropriate to the culture in which the study is to be conducted, is to be strongly encouraged. Ease of between-study comparisons would be enhanced if investigators were to consistently report both rates of olfactory impairment (based on well-defined criteria) and aggregated olfactory test score data (e.g., as expressed by group specific means ± standard deviation) as the latter measure does not necessarily easily convert to the former metric (i.e., prevalence of impairment).

As clinicians who routinely evaluate cognition, we are struck by the parallels between this activity and the conduct and interpretation of olfactory testing. The analogy holds in the context of TBI specifically. For both cognition and olfaction, the absence of symptoms cannot be relied upon to indicate intact functioning, or complaints to predict impairment on objective testing. Brevity of a screening instrument, while “convenient,” usually comes at the cost of reduced sensitivity but some testing is (almost always) better than none at all. Age, gender, and many extraneous factors may affect performance and, in the absence of a previous “premorbid” assessment, the etiological significance of a single abnormal test result may be difficult to determine, although the clinical context (and pre-test probability of impairment) are clearly relevant. Patients’ occupational and social responsibilities (viz. the cognitive or olfactory functioning challenges associated) might sensibly dictate who to prioritize for testing but such a strategy needs a background level of awareness as to the possibility of problems. We hope that this systematic review will make some contribution toward raising the awareness level in relation to olfactory dysfunction following TBI.

## Conflict of Interest Statement

The authors declare that the research was conducted in the absence of any commercial or financial relationships that could be construed as a potential conflict of interest.

## References

[1] Sumner D Post traumatic anosmia. Brain (1964) 87:107–20 10.1093/brain/87.1.107 14156077

[2] Doty RL Shaman P Dann M Development of the University of Pennsylvania Smell Identification test: a standardized microencapsulated test of olfactory function. Physiol Behav (1984) 32:489–502 10.1016/0031-9384(84)90269-5 6463130

[3] Kobal G Hummel T Sekinger B Barz S Roscher S Wolf SR ‘Sniffin’ Sticks’: screening of olfactory performance. Rhinology (1996) 34:222–6 9050101

[4] Parma V Straulino E Zanatto D Cantagallo A Tirindelli R Castiello U Implicit olfactory abilities in traumatic brain injured patients. J Clin Exp Neuropsychol (2012) 34:977–88 10.1080/13803395.2012.711811 22905854

[5] Neumann D Zupan B Babbage DR Radnovich AJ Tomita M Hammond F Affect recognition, empathy, and dysosmia after traumatic brain injury. Arch Phys Med Rehabil (2012) 93:1414–20 10.1016/j.apmr.2012.03.009 22446155

[6] Charland-Verville V Lassonde M Frasnelli J Olfaction in athletes with concussion. Am J Rhinol Allergy (2012) 26:222–6 10.2500/ajra.2012.26.3769 22643951

[7] Welge-Lussen A Hilgenfeld A Meusel T Hummel T Long-term follow-up of posttraumatic olfactory disorders. Rhinology (2012) 50:67–72 10.4193/Rhino11.141 22469607

[8] Gerami H Nemati S Abbaspour F Banan R Brain single photon emission computed tomography in anosmic subjects after closed head trauma. Acta Med Iran (2011) 49:13–7 21425064

[9] Sigurdardottir S Jerstad T Andelic N Roe C Schanke AK Olfactory dysfunction, gambling task performance and intracranial lesions after traumatic brain injury. Neuropsychology (2010) 24:504–13 10.1037/a0018934 20604624

[10] Vent J Koenig J Hellmich M Huettenbrink KB Damm M Impact of recurrent head trauma on olfactory function in boxers: a matched pairs analysis. Brain Res (2010) 1320:1–6 10.1016/j.brainres.2010.01.007 20064489

[11] Fortin A Lefebvre MB Ptito M Traumatic brain injury and olfactory deficits: the tale of two smell tests! Brain Inj (2010) 24:27–33 10.3109/02699050903446815 20001480

[12] Haxel BR Grant L Mackay-Sim A Olfactory dysfunction after head injury. J Head Trauma Rehabil (2008) 23:407–13 10.1097/01.HTR.0000341437.59627.ec 19033834

[13] Rombaux P Mouraux A Bertrand B Nicolas G Duprez T Hummel T Retronasal and orthonasal olfactory function in relation to olfactory bulb volume in patients with posttraumatic loss of smell. Laryngoscope (2006) 116:901–5 10.1097/01.MLG.0000195291.36641.1E 16735894

[14] Sandford AA Davidson TM Herrera N Gilbert P Magit AE Haug K Olfactory dysfunction: a sequela of pediatric blunt head trauma. Int J Pediatr Otorhinolaryngol (2006) 70:1015–25 10.1016/j.ijporl.2005.10.013 16360887

[15] Green P Rohling ML Iverson GL Gervais RO Relationships between olfactory discrimination and head injury severity. Brain Inj (2003) 17:479–96 10.1080/0269905031000070242 12745704

[16] de Kruijk JR Leffers P Menheere PP Meerhoff S Rutten J Twijnstra A Olfactory function after mild traumatic brain injury. Brain Inj (2003) 17:73–8 10.1080/0269905021000010221 12519649

[17] Callahan CD Hinkebein JH Assessment of anosmia after traumatic brain injury: performance characteristics of the University of Pennsylvania Smell Identification test. J Head Trauma Rehabil (2002) 17:251–6 10.1097/00001199-200206000-00006 12086578

[18] Fujii M Fukazawa K Takayasu S Sakagami M Olfactory dysfunction in patients with head trauma. Auris Nasus Larynx (2002) 29:35–40 10.1016/S0385-8146(01)00118-3 11772488

[19] Green P Iverson GL Effects of injury severity and cognitive exaggeration on olfactory deficits in head injury compensation claims. NeuroRehabilitation (2001) 16:237–43 11790910

[20] Yousem DM Geckle RJ Bilker WB Kroger H Doty RL Posttraumatic smell loss: relationship of psychophysical tests and volumes of the olfactory bulbs and tracts and the temporal lobes. Acad Radiol (1999) 6:264–72 10.1016/S1076-6332(99)80449-8 10228615

[21] Geisler MW Schlotfeldt CR Middleton CB Dulay MF Murphy C Traumatic brain injury assessed with olfactory event-related brain potentials. J Clin Neurophysiol (1999) 16:77–86 10.1097/00004691-199901000-00008 10082095

[22] Callahan CD Hinkebein J Neuropsychological significance of anosmia following traumatic brain injury. J Head Trauma Rehabil (1999) 14:581–7 10.1097/00001199-199912000-00006 10671703

[23] Doty RL Yousem DM Pham LT Kreshak AA Geckle R Lee WW Olfactory dysfunction in patients with head trauma. Arch Neurol (1997) 54:1131–40 10.1001/archneur.1997.00550210061014 9311357

[24] Levin HS High WM Eisenberg HM Impairment of olfactory recognition after closed head injury. Brain (1985) 108:579–91 10.1093/brain/108.3.579 4041775

[25] Drummond M Douglas J Olver J ‘If I haven’t got any smell. I’m out of work’: consequences of olfactory impairment following traumatic brain injury. Brain Inj (2013) 27:332–45 10.3109/02699052.2012.750743 23438353

[26] Ruff RL Riechers RG Wang XF Piero T Ruff SS For veterans with mild traumatic brain injury, improved posttraumatic stress disorder severity and sleep correlated with symptomatic improvement. J Rehabil Res Dev (2012) 49:1305–20 10.1682/JRRD.2011.12.0251 23408213

[27] Joung YI Yi HJ Lee SK Im TH Cho SH Ko Y Posttraumatic anosmia and ageusia: Incidence and recovery with relevance to the hemorrhage and fracture on the frontal base. J Korean Neurosurg Soc (2007) 42:1–5

[28] Hirsch AR Wyse JP Posttraumatic dysosmia: central vs. peripheral. J Neurol Orthop Med Surg (1993) 14:152–5

